# The effect of accompanying anxiety and depression on patients with different vestibular syndromes

**DOI:** 10.3389/fnagi.2023.1208392

**Published:** 2023-08-01

**Authors:** Shuai Feng, Jian Zang

**Affiliations:** Department of Otolaryngology, The First Hospital of China Medical University, Shenyang, China

**Keywords:** vertigo, vestibular syndrome, anxiety, depression, dizziness handicap inventory

## Abstract

**Objective:**

This study aims to investigate the situation of vertigo disorder combined with anxiety and depression in patients with different types of vestibular syndrome.

**Methods:**

A total of 330 patients with vertigo in otolaryngology outpatient department were selected, and clinical information such as age, gender, and scores of Dizziness handicap inventory (DHI), Generalized anxiety disorder-7 (GAD-7), and Patient Health Questionnaire-9 (PHQ-9) were collected. Analyzed the differences among acute vestibular syndrome (AVS), episodic vestibular syndrome (EVS) and chronic vestibular syndrome (CVS) in terms of age, gender, comorbid anxiety and depression, and the multivariate ordered logistic regression analysis was used to evaluate the relationship between the above factors and the degree of vertigo disorder.

**Results:**

The three types of vestibular syndrome had no significant difference in age composition, sex composition, anxiety and depression. There was no significant difference in the probability of anxiety and depression among vertigo patients of different ages and genders. The total score of vertigo disorder and each sub-item score were higher in patients with anxiety and depression. Patients with anxiety mainly manifested in EVS and CVS, while patients with depression mainly manifested in EVS and AVS. The probability of increased vertigo in anxious patients was 4.65 times that of non-anxious patients, and the probability of increased vertigo in depressed patients was 3.49 times that of non-depressed patients. Age and gender had no statistically significant effect on the degree of vertigo. In patients with EVS, anxiety and depression had a significant effect on the degree of vertigo; in patients with CVS, anxiety had a significant effect on the degree of vertigo, but depression had no significant effect.

**Conclusion:**

Age and gender do not significantly affect the degree of vertigo disorder and mental state in various vestibular syndromes. Instead, anxiety and depression are the risk factors for aggravating the degree of vertigo disorder, and manifest differently in each type of vestibular syndrome. Therefore, it is necessary to use a quick scale tool to conduct a standardized screening of the psychological status of patients with vertigo.

## 1. Introduction

Vertigo, a common clinical symptom, is the most prominent manifestation of vestibular disorders. Dysfunction of the vestibular system can lead to a variety of symptoms, from vertigo, vision and balance problems, to mood, memory, and self-perception problems. Large population-based studies have shown that dizziness and vertigo affect approximately 15% to over 20% of adults annually. Vestibular vertigo accounts for approximately 1/4 of dizziness complaints and can cause unilateral or bilateral deterioration and loss of vestibular function, which can significantly impact work and living activities (Quimby et al., 2018). The prevalence of vertigo increases with age and is approximately 2–3 times higher in women than in men. An epidemiological study in the United States showed that the number of patients with dizziness and balance disorders as the chief complaint was about 33 million, for an annual prevalence of 14.8% (Kerber et al., 2017).

The 2015 International Classification of Vestibular Disorders (ICVD) divides vestibular disorders into acute vestibular syndrome (AVS), episodic vestibular syndrome (EVS), and chronic vestibular syndrome (CVS) (Bisdorff et al., 2015). The generally accepted definition of AVS is sudden-onset, continuous vertigo lasting over 24 h and accompanied by nausea/vomiting, exercise intolerance, and gait instability; acute unilateral peripheral vestibular lesions are considered the most common cause, including diseases such as acute unilateral vestibulopathy (AUVP)/vestibular neuritis (VN) and sudden deafness with vertigo. EVS refers to recurrent vestibular disorders that are symptomatic during episodes with remission between episodes, and usually includes some transient vestibular system dysfunction (e.g., nystagmus, drop attacks). Signs and symptoms suggestive of cochlear or central nervous system dysfunction may also be present, including diseases such as benign paroxysmal positional vertigo (BPPV), Menière’s disease (MD), vestibular migraine (VM), transient ischemic attack (TIA), vestibular paroxysmia (VP). CVS is a group of clinical syndromes characterized by chronic dizziness, vertigo, or instability. It lasts from several months to several years, usually with persistent vestibular system dysfunction (visual oscillations, nystagmus, gait instability), including diseases such as bilateral vestibulopathy (BVP) and persistent postural perceptual dizziness (PPPD). The symptoms of CVS can be a gradual progression of deterioration, or they can manifest as persistent symptoms of stable but incomplete recovery from AVS or EVS (Trinidade et al., 2023).

About 20–50% of patients with vertigo and balance disorders have concomitant psychiatric disorders (McKenna et al., 1991; Best et al., 2009; Al-Rawashdeh et al., 2019). Due to the long disease duration, many vertigo patients suffer from anxiety, and concomitant psychiatric disorders such as anxiety and depression have become commonplace among vertigo patients. The prevalence of anxiety and depression among patients with symptoms of vertigo has been reported to be 18 and 11%, respectively (Kim et al., 2016). Ketola et al. (2007) found that 20% of a random sample of 100 vertigo patients reported symptoms of depression. Eckhardt-Henn et al. (2003) conducted neuro-otological examination, vestibular tests, and psychiatric examination and administered evaluations and questionnaires to 129 patients with vertigo accompanied by psychiatric disorders and found a higher prevalence of anxiety (41%) than depression (15%). An analysis of 621 patients in a vertigo clinic by van Leeuwen et al. (2017) found that the most common secondary diagnosis in patients with vertigo was anxiety (∼50.1%), which was found primarily in conjunction with peripheral vestibular disorders such as BPPV, vestibular neuritis, and Ménière disease. Grunfeld et al. (2003) found a higher incidence of depression and anxiety among vertigo patients than in normal individuals using the HADS instrument. A cross-sectional study of vertigo patients by Roh et al. (2017) concluded that the emotional state of vertigo patients may be associated with the persistence of vertigo symptoms and that vertigo-induced psychiatric distress may contribute to a prolonged duration of disease; they recommend timely screening for psychiatric disorders.

Brandt and Dieterich (2020) suggested that damage to the vestibular system is a central factor in the development of mood disorders such as anxiety and depression, and that vestibular hyperfunction (acute excitation or acute vestibular tone imbalance) or hypofunction (chronic vestibular loss) under certain conditions can lead to the development of mood disorders. Furman et al. (2005) and Goddard et al. (2008) found that the onset and interaction between vestibular disorders and psychiatric disorders is associated with overlapping central nervous system transmission of the vestibular and mood information pathways. The vestibular nucleus, which controls vertigo, has many nerve fiber projections with mood-related nuclei such as the parabrachial nuclei, the locus coeruleus, and the dorsal raphe nuclei, and also interacts with the frontal lobe, hippocampus, and dentate gyrus. This affects the release of catecholamines (dopamine, norepinephrine), 5-HT, and other neurotransmitters, causing dysfunction in these mood-related regions and affecting the development of anxiety and depression (Furman et al., 2005; Goddard et al., 2008; Bednarczuk et al., 2018; Hilber, 2022).

By far the most common psychiatric comorbidity in vertigo patients is anxiety and/or depression, which is primarily associated with gender, age, duration of vertigo, frequency of vertigo episodes, and degree of severity of vertigo. The psychiatric state of the patient plays an important role in the prognosis of vertigo disease (Tschan et al., 2011) but is often neglected by clinicians, which affects the efficacy of vertigo treatment. Both psychological and physical factors must be considered in the clinical treatment of vertigo, and the psychological status of patients warrants increased attention. In addition, intervention with anxiolytic and antidepressant medications and psychotherapy should be considered when determining the best treatment plan. In the present study, we analyzed the general condition and etiology of each type of vestibular syndrome among vertigo outpatients, and also conducted an assessment of anxiety and depression in the patients with the aim of enabling physicians to improve outcomes through more comprehensive communication, psychological guidance, and medication for vertigo patients. The present study analyzed the relationship between vestibular syndrome and anxiety and depression in different genders, age groups, and types of vestibular syndrome.

## 2. Materials and methods

### 2.1. Source of cases

A cross-sectional study design was adopted. The study was approved by the Ethics Committee. The study population consisted of 386 patients with vertigo who visited the otolaryngology department of the first hospital of China medical university between March 2022 and October 2022. A total of 330 cases were collected after applying the inclusion and exclusion criteria.

### 2.2. Inclusion criteria

Chief complaint of vertigo or dizziness, meets the diagnostic criteria for each disorder, and relatively complete medical records.

### 2.3. Exclusion criteria

Patients with major psychiatric disorders or cognitive dysfunction and patients with vertigo clearly caused by other disorders, such as cranial lesions, middle ear lesions, inner ear malformations, internal auditory tract lesions, drug effects, and other medical disorders.

### 2.4. Diagnostic standards for classification of vertigo

(1) Vestibular syndrome was classified into three categories in accordance with the 2015 ICVD (Bisdorff et al., 2015): AVS, EVS, and CVS.
(2) Benign paroxysmal positional vertigo was diagnosed in accordance with the 2015 diagnostic criteria for BPPV formulated by the Bárány Society (von Brevern et al., 2015).
(3) Vestibular migraine was diagnosed in accordance with the 2022 diagnostic criteria for VM formulated by the Bárány Society and the International Headache Society (Lempert et al., 2022).
(4) Menière’s disease was diagnosed in accordance with the 2015 diagnostic criteria for MD formulated by the Bárány Society, the Japan Society for Equilibrium Research, the European Academy of Otology and Neurotology, the Equilibrium Committee of the American Academy of Otolaryngology-Head and Neck Surgery, and the Korean Balance Society (Ihler et al., 2022).
(5) Acute unilateral vestibulopathy/vestibular neuritis were diagnosed in accordance with the 2022 diagnostic criteria for AUVP/VN formulated by the Bárány Society (Strupp et al., 2022).
(6) Persistent postural perceptual dizziness was diagnosed in accordance with the 2017 consensus document of the committee for the Bárány Society (Staab et al., 2017).
(7) Sudden hearing loss with vertigo was diagnosed in accordance with the 2019 American Academy of Otolaryngology-Head and Neck Surgery Clinical Practice Guideline for Sudden Hearing Loss (Chandrasekhar et al., 2019).
(8) Unilateral vestibular hypofunction (UVH) was diagnosed in accordance with the 2022 Vestibular Rehabilitation for Peripheral Vestibular Hypofunction: An Updated Clinical Practice Guideline From the Academy of Neurologic Physical Therapy of the American Physical Therapy Association (Hall et al., 2022).
(9) Bilateral vestibulopathy was diagnosed in accordance with the 2017 diagnostic criteria for BVP developed by the Classification Committee of the Bárány Society (Strupp et al., 2017).
(10) Unspecified diagnosis: failure to meet the diagnostic criteria due to lack of clinical evidence, including history of suspected VP, posterior circulation ischemic stroke, and TIA.

### 2.5. Evaluation of vertigo disorder

The DHI is a widely used self-report questionnaire for patients with dizziness or vertigo that has been translated into 14 languages and is widely validated (Mutlu and Serbetcioglu, 2013). The DHI consists of 25 items and 4 measurements: the total score and 3 sub-scores (emotional subdomain, DHI-E; functional subdomain, DHI-F; physical subdomain, DHI-P). The total DHI score ranges between 0 and 100 and is used to holistically assess the subjective severity of vertigo symptoms (mild, 0–30; moderate, 31–60; severe, >60).

### 2.6. Psychological evaluation

The GAD-7 is a simple and effective assessment tool for identifying generalized anxiety disorder and has good sensitivity and specificity for screening anxiety (89% sensitivity, 82% specificity) when the GAD-7 score is ≥10 (Spitzer et al., 2006). Therefore, a GAD-7 score ≥10 was defined as anxiety in this study. GAD-7 scores can be divided into three ranges: 5–9, 10–14, and 15–21, representing mild, moderate, and severe anxiety disorder, respectively.

The PHQ-9 is an important tool for assessing depression and its severity and is widely used for the screening of psychiatric disorders. A score of 10 or more exhibits good sensitivity and specificity (88% sensitivity, 85% specificity) (Levis et al., 2019, 2020). Therefore, a PHQ-9 score ≥10 was defined as depression in this study. PHQ-9 scores can be divided into four ranges: 5–9, 10–14, 15–19, and 20–27, representing mild, moderate, moderate-severe, and major depression, respectively.

### 2.7. Case data collection and processing

Collected information on the patient’s general condition, type of vertigo symptoms, duration, precipitating factors, past medical history, concomitant symptoms, ancillary examinations, and disease diagnosis.

Under the guidance of specialists, patients completed the DHI questionnaire, the GAD-7 anxiety screening scale, and the PHQ-9 depression screening scale.

Two independent researchers reviewed and validated the completed questionnaires and entered the collected and organized data into the Epidate 3.1 database.

### 2.8. Data analysis

Data were imported into SPSS 22.0 statistical software for analysis. Descriptive statistic included mean and standard deviation (SD), and proportion. The *t*-test were used for analyzing measurement data, and the chi-square test and fisher’s exact test were used for analyzing count data. The relationships between influencing factors were analyzed using multivariate ordered logistic regression analysis. Differences with *p* < 0.05 were considered statistically significant.

## 3. Results

The baseline characteristics of the vertigo patients are shown in Table 1.

**TABLE 1:** Clinical data of participants with vestibular disorder.

Characteristic	Full sample (*n* = 330)	AVS (*n* = 42)	EVS (*n* = 236)	CVS (*n* = 52)
**Age**				
Average age^b^	53.18 ± 14.40	53.71 ± 15.18	52.26 ± 14.95	56.94 ± 9.87
<60^a^	205 (62.12)	24 (57.14)	151 (63.98)	30 (57.69)
≥60^a^	125 (37.88)	18 (42.86)	85 (36.02)	22 (42.31)
**Gender^a^**				
Male	94 (28.48)	11 (26.19)	68 (28.81)	15 (28.85)
Female	236 (71.52)	31 (73.81)	168 (71.19)	37 (71.15)
**Complications^a^**				
Deafness	58 (17.58)	8 (19.05)	40 (16.95)	10 (19.23)
Tinnitus	125 (37.88)	16 (38.10)	88 (37.29)	21 (40.38)
Headache	102 (30.91)	13 (30.95)	65 (27.54)	24 (46.15)
**Past history^a^**				
Cardiovascular and cerebrovascular diseases	123 (37.27)	13 (30.95)	85 (36.02)	25 (48.08)
Diabetes	29 (8.79)	8 (19.05)	19 (8.05)	2 (3.85)
Neurological disorders	19 (5.76)	3 (7.14)	12 (5.08)	4 (7.69)
Sleep disturbances	33 (10.00)	6 (14.29)	18 (7.63)	9 (17.31)
GAD-7 (anxiety)^a^*				
Total < 10	273 (87.50)	36 (92.31)	194 (87.39)	43 (84.31)
Total ≥ 10	39 (12.50)	3 (7.69)	28 (12.61)	8 (15.69)
PHQ-9 (depression)^a^*				
Total < 10	271 (88.27)	35 (89.74)	194 (88.18)	42 (87.50)
Total ≥ 10	36 (11.73)	4 (10.26)	26 (11.82)	6 (12.50)
**Vertigo disorder (DHI)**				
Total^b^	43.36 ± 24.69	41.52 ± 26.28	41.74 ± 24.09	52.33 ± 24.11
Physical^b^	13.65 ± 7.42	13.19 ± 9.70	13.22 ± 6.84	16.00 ± 7.56
Emotional^b^	11.68 ± 9.71	10.00 ± 8.33	11.37 ± 9.71	14.46 ± 10.38
Functional^b^	18.60 ± 11.46	18.29 ± 11.43	17.73 ± 11.54	22.81 ± 10.34
Mild^a^	116 (35.15)	19 (45.24)	83 (35.17)	14 (26.92)
Moderate^a^	130 (39.39)	13 (30.95)	101 (42.80)	16 (30.77)
Severe^a^	84 (25.45)	10 (23.81)	52 (22.03)	22 (42.31)

^*a*^*n* (%).

^*b*^Mean ± SD.

*The full sample *n* = 330, where the GAD-7 score has 18 missing values and the PHQ-9 score has 23 missing values.

The mean age of the 330 vertigo patients was 53.18 years (SD: 14.40 years), and 37.88% were aged ≥60 years. The male-to-female ratio of the patients was 1:2.51. Of these patients, 12.50% were anxious and 11.73% were depressed. With respect to syndrome typing, AVS accounted for 12.72%, EVS accounted for 71.52%, and CVS accounted for 15.76%. The mean DHI score was 43.36 (SD: 24.69), with 35.15% of mild disorder, 39.39% of moderate disorder, and 25.45% of severe disorder.

There was no significant differences in age structure, gender ratio, comorbid anxiety, or comorbid depression among patients with AVS, EVS, and CVS (Table 2).

**TABLE 2:** Age, gender and manifestations of anxiety and depression in patients with different syndromes.

Characteristic	AVS (*n* = 42)	EVS (*n* = 236)	CVS (*n* = 52)	χ^2^	*P*
Age				1.224	0.542
<60 (*n* = 205)	24 (57.14)	151 (63.98)	30 (57.69)		
≥60 (*n* = 125)	18 (42.86)	85 (36.02)	22 (42.31)		
Gender				0.124	0.940
Male (*n* = 94)	11 (26.19)	68 (28.81)	15 (28.85)		
Female (*n* = 236)	31 (73.81)	168 (71.19)	37 (71.15)		
GAD-7*				1.228	0.585
Total < 10 (*n* = 273)	36 (92.31)	194 (87.39)	43 (84.31)		
Total ≥ 10 (*n* = 39)	3 (7.69)	28 (12.61)	8 (15.69)		
PHQ-9*				0.130	0.961
Total < 10 (*n* = 271)	35 (89.74)	194 (88.18)	42 (87.50)		
Total ≥ 10 (*n* = 36)	4 (10.26)	26 (11.82)	6 (12.50)		

*In the GAD-7 score, there were 18 missing values in the full sample, 3 missing values in AVS, 14 missing values in EVS, 1 missing value in CVS. In the PHQ-9 score, there were 23 missing values in the full sample, 3 missing values in AVS, 16 missing values in EVS, 4 missing values in CVS.

The probability of presenting with anxiety and depression did not differ significantly among vertigo patients of different ages and genders (Tables 3, 4).

**TABLE 3:** Combined anxiety in patients with vertigo of different ages and genders.

	Full sample (*n* = 312)		*P*	AVS (*n* = 39)		*P*	EVS (*n* = 222)		*P*	CVS (*n* = 51)		*P*
	GAD7 < 10	≥10		GAD7 < 10	≥10		GAD7 < 10	≥10		GAD7 < 10	≥10	
Age			0.894			1.000			0.945			1.000
<60	172 (63.00)	25 (64.10)		22 (61.11)	2 (66.67)		126 (64.95)	18 (64.29)		24 (55.81)	5 (62.50)	
≥60	101 (37.00)	14 (35.90)		14 (38.89)	1 (33.33)		68 (35.05)	10 (35.71)		19 (44.19)	3 (37.50)	
Gender			0.704			0.127			0.930			1.000
Male	76 (27.84)	12 (30.77)		7 (19.44)	2 (66.67)		57 (29.38)	8 (28.57)		12 (27.91)	2 (25.00)	
Female	197 (72.16)	27 (69.23)		29 (80.56)	1 (33.33)		137 (70.62)	20 (71.43)		31 (72.09)	6 (75.00)	

Chi-square test was used to analysis for full sample and EVS, and Fisher’s exact test was used to analysis for AVS and CVS.

**TABLE 4:** Combined depression in patients with vertigo of different ages and genders.

	Full sample (*n* = 307)		*P*	AVS (*n* = 39)		*P*	EVS (*n* = 220)		*P*	CVS (*n* = 48)		*P*
	PHQ9 < 10	≥10		PHQ9 < 10	≥10		PHQ9 < 10	≥10		PHQ9 < 10	≥10	
Age			0.645			1.000			0.630			1.000
<60	170 (62.73)	24 (66.67)		21 (60.00)	3 (75.00)		125 (64.43)	18 (69.23)		24 (57.14)	3 (50.00)	
≥60	101 (37.27)	12 (33.33)		14 (40.00)	1 (25.00)		69 (35.57)	8 (30.77)		18 (42.86)	3 (50.00)	
Gender			0.718			1.000			0.798			1.000
Male	75 (27.68)	11 (30.56)		8 (22.86)	1 (25.00)		55 (28.35)	8 (30.77)		12 (28.57)	2 (33.33)	
Female	196 (72.32)	25 (69.44)		27 (77.14)	3 (75.00)		139 (71.65)	18 (69.23)		30 (71.43)	4 (66.67)	

Chi-square test was used to analysis for full sample and EVS, and Fisher’s exact test was used to analysis for AVS and CVS.

When patients with various forms of vertigo were analyzed, a significant difference in the total DHI score among all patients was found between those with and without anxiety (*P* < 0.001), and there were differences in physical symptoms (*P* = 0.020), emotional state (*P* < 0.001), and social functioning (*P* < 0.001). In EVS, there was a significant difference in total DHI score between those with and without anxiety (*P* < 0.001), and there were differences in physical symptoms (*P* = 0.021), emotional state (*P* < 0.001), and social functioning (*P* < 0.001). In CVS, there was a significant difference in total DHI score between those with and without anxiety (*P* = 0.018), which was primarily manifested in a difference in emotional state (*P* = 0.023) (Table 5).

**TABLE 5:** Differences of anxiety in various forms of vertigo disorder.

	Full sample (*n* = 312)		*P*	AVS (*n* = 39)		*P*	EVS (*n* = 222)		*P*	CVS (*n* = 51)		*P*
	GAD7 < 10	≥10		GAD7 < 10	≥10		GAD7 < 10	≥10		GAD7 < 10	≥10	
DHI Total	40.99 ± 24.27	61.42 ± 22.75	**<0.001**	41.67 ± 26.84	56.67 ± 33.01	0.365	39.13 ± 23.57	59.11 ± 22.80	**<0.001**	48.93 ± 24.10	71.00 ± 18.94	**0.018**
Physical	13.37 ± 7.48	16.36 ± 7.33	**0.020**	13.83 ± 10.09	10.00 ± 9.17	0.529	12.85 ± 6.77	16.07 ± 7.35	**0.021**	15.35 ± 7.84	19.75 ± 5.29	0.135
Emotional	10.51 ± 9.25	20.36 ± 9.41	**<0.001**	9.67 ± 7.89	18.67 ± 12.22	0.075	10.05 ± 9.29	20.00 ± 9.11	**<0.001**	13.26 ± 9.82	22.25 ± 10.61	**0.023**
Functional	17.55 ± 11.37	26.15 ± 10.14	**<0.001**	18.17 ± 11.55	26.67 ± 12.86	0.231	16.55 ± 11.35	25.29 ± 10.92	**<0.001**	21.53 ± 10.66	29.00 ± 6.23	0.062

The bold values indicate mean *p* < 0.05, these values were considered statistically significant.

When patients with various forms of vertigo were analyzed, a significant difference in the total DHI score among all patients was found between those with and without depression (*P* < 0.001), and there were differences in physical symptoms (*P* = 0.006), emotional state (*P* < 0.001), and social functioning (*P* < 0.001). In EVS, there was a significant difference in total DHI score between those with and without depression (*P* = 0.008), and there were differences in physical symptoms (*P* = 0.030), emotional state (*P* = 0.041), and social functioning (*P* = 0.030). In AVS, there was a significant difference in total DHI score between those with and without depression (*P* = 0.005), which was primarily manifested in difference in emotional state (*P* < 0.001) and social functioning (*P* = 0.006) (Table 6).

**TABLE 6:** Differences of depression in various forms of vertigo disorder.

	Full sample (*n* = 307)		*P*	AVS (*n* = 39)		*P*	EVS (*n* = 220)		*P*	CVS (*n* = 48)		*P*
	PHQ9 < 10	≥10		PHQ9 < 10	≥10		PHQ9 < 10	≥10		PHQ9 < 10	≥10	
DHI Total	41.28 ± 24.62	58.72 ± 23.69	**<0.001**	38.86 ± 25.59	77.50 ± 11.36	**0.005**	39.60 ± 24.09	53.00 ± 23.67	**0.008**	51.20 ± 24.50	71.00 ± 20.39	0.066
Physical	13.32 ± 7.57	17.00 ± 6.30	**0.006**	12.80 ± 10.07	20.00 ± 6.73	0.174	12.79 ± 6.89	15.92 ± 6.49	**0.030**	16.19 ± 7.74	19.67 ± 4.27	0.290
Emotional	10.92 ± 9.67	17.00 ± 9.90	**<0.001**	8.91 ± 7.25	23.00 ± 8.25	**<0.001**	10.64 ± 9.86	14.85 ± 9.25	**0.041**	13.90 ± 10.10	22.33 ± 11.41	0.066
Functional	17.81 ± 11.52	24.61 ± 10.46	**<0.001**	17.14 ± 11.15	33.50 ± 1.92	**0.006**	16.96 ± 11.61	22.23 ± 10.90	**0.030**	22.29 ± 10.54	29.00 ± 7.35	0.140

The bold values indicate mean *p* < 0.05, these values were considered statistically significant.

The relationship between each influencing factor and the severity of vertigo was determined using multivariate ordered logistic regression analysis (DHI “mild/moderate/severe” severity was the ordered classification outcome, model fit *p* < 0.001 indicated effective establishment of the regression equation, test of parallel lines *p* = 0.776 > 0.05 indicated equivalence to mild/moderate/severe). Age ≥60 or <60 years and gender had no statistically significant effects on the severity of vertigo.

Among all patients, patients with anxiety were 4.65 times more likely to have severe vertigo than patients without anxiety, and patients with depression were 3.49 times more likely to have severe vertigo than patients without depression.

**FIGURE 1:**
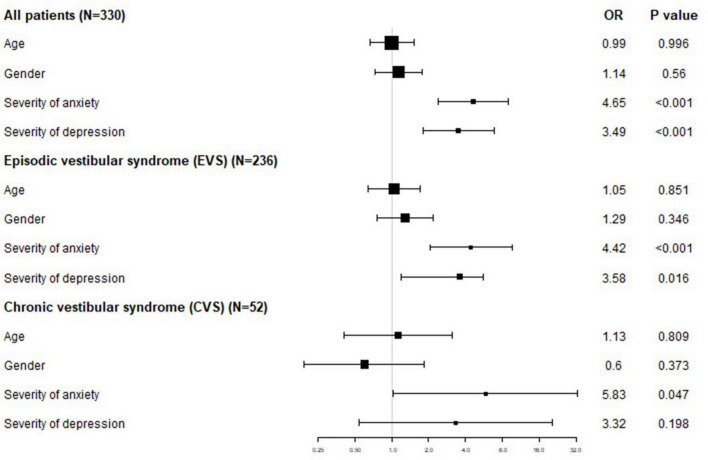
Influence of age, gender, anxiety, and depression on vertigo degree.

Among AVS patients, anxiety and depression had no statistically significant effect on the severity of vertigo. Among EVS patients, patients with anxiety were 4.42 times more likely to have severe vertigo than patients without anxiety, and patients with depression were 3.58 times more likely to have severe vertigo than patients without depression. Among CVS patients, patients with anxiety were 5.83 times more likely to have severe vertigo than patients without anxiety, and depression had no statistically significant effect on the severity of vertigo (Table 7 and Figure 1).

**TABLE 7:** Influence of various factors on vertigo degree in patients with different vestibular syndromes.

	Full sample (*n* = 330)		AVS (*n* = 42)		EVS (*n* = 236)		CVS (*n* = 52)	
	*OR*	*P*	*OR*	*P*	*OR*	*P*	*OR*	*P*
Age	0.999 (0.66, 1.51)	0.996	0.63 (0.20, 1.997)	0.435	1.05 (0.64, 1.72)	0.851	1.13 (0.41, 3.14)	0.809
Gender	1.14 (0.73, 1.78)	0.560	1.63 (0.44, 6.07)	0.463	1.29 (0.76, 2.18)	0.346	0.60 (0.19, 1.85)	0.373
GAD-7	**4.65 (2.40, 9.03)**	**<0.001**	4.45 (0.44, 45.19)	0.206	**4.42 (2.04, 9.58)**	**<0.001**	**5.83 (1.03, 33.04)**	**0.047**
PHQ-9	**3.49 (1.79, 6.82)**	**<0.001**	−^#^	−^#^	**3.58 (1.19, 5.57)**	**0.016**	3.32 (0.54, 20.53)	0.198

^#^The value is abnormality. The bold values indicate mean *p* < 0.05, these values were considered statistically significant.

## 4. Discussion

In recent years, comorbid psychological and physical symptoms in vertigo patients has gained interest among clinicians. The coexistence of vestibular and psychiatric disorders has been repeatedly described in the literature (Best et al., 2009; Lahmann et al., 2015; Limburg et al., 2018; Bronstein and Dieterich, 2019; Toupet et al., 2019; Özdilek et al., 2019; Kısabay Ak et al., 2022), with anxiety and depression cited as the primary factors. Many studies have reported a significantly higher prevalence of comorbid peripheral vertigo and anxiety and depression than in the general population (Brandt and Dieterich, 2020). Kim et al. (2016) found a higher prevalence of psychiatric disorders such as depression and generalized anxiety in patients with BPPV compared to the general population (Kozak et al., 2018). A prospective study by Özdilek et al. (2019) showed that Beck Anxiety Inventory (BAI) scores were higher in patients with BPPV than in control individuals, indicating higher levels of anxiety in patients with BPPV. Psychological symptoms such as anxiety and depression are common in patients with vertigo regardless of its primary cause (Ketola et al., 2015; Zhai et al., 2016; Limburg et al., 2018; Balci and Akdal, 2020; Kısabay Ak et al., 2022). Our study suggests that the association between vertigo and anxiety and depression is very strong. In our sample, 12.50% of vertigo patients had comorbid anxiety and 11.73% of vertigo patients had comorbid depression.

The potential mechanisms underlying the connection between vertigo and psychiatric disorders remain unclear. Neural circuits associated with the vestibular nervous system and psychiatric disorders such as anxiety and depression have been found to be interrelated (Bednarczuk et al., 2018; Bronstein and Dieterich, 2019; Decker et al., 2019; Toupet et al., 2019; Brandt and Dieterich, 2020), and there is growing evidence to support that psychological factors influence vertigo episodes and response to treatment in a complex manner (Bigelow et al., 2016; Wei et al., 2018; Brandt and Dieterich, 2020; Ogihara et al., 2022). Two large-scale, population-based retrospective studies have confirmed that patients with anxiety and depression have a higher risk of developing BPPV than healthy individuals, with hazard ratios of 2.52 and 1.79, respectively (Chen et al., 2016; Hsu et al., 2019). Bronstein and Dieterich (2019) found that the long-term prognostic outcome of vestibular neuritis is largely dependent on psychophysiological and psychological factors. Ogihara et al. (2022) suggest that psychological factors may influence the rehabilitation of vestibular balance disorders, leading to long-term vertigo or dizziness. Anxiety and depression can significantly reduce the efficacy of the initial canalith repositioning treatment in patients with BPPV and lead to a pronounced risk of relapse within 6 months after treatment (Wei et al., 2018). Kısabay Ak et al. (2022) investigated risk factors for reduced treatment response in patients with comorbid anxiety and depressive VM, and found that VM patients who do not respond to prophylactic medication should be examined for the presence of comorbid psychiatric disorders and additional treatment strategies should be implemented. Monzani et al. (2006) found that BPPV patients had significantly higher rates of negative life events, objective negative affect and poor control, anxiety, depression, somatization levels, and obsessive-compulsive attitudes than controls in the year prior to the vertigo episode, suggesting that emotional stress may be a trigger for vestibular dysfunction. A prospective study found that the probability of RD increased with increasing DHI-E scores and that mood disorders may be an important risk factor for RD (Martellucci et al., 2016). The consensus diagnostic criteria for PPPD formulated by the Bárány Society state that anxiety and depression are risk factors for PPPD (Staab et al., 2017). In this study, we found that patients with comorbid anxiety or depression exhibited more severe vertigo and impacts on somatic symptoms, emotional state, and social function than patients without anxiety and/or depression. Multivariate ordered logistic regression analysis showed that comorbid anxiety or depression was an important risk factor that could significantly exacerbate vertigo. When different types of vestibular syndrome were analyzed separately, the association between EVS and anxiety or depression was most pronounced, with EVS exacerbated by either anxiety or depression. In patients with CVS, anxiety also significantly exacerbated vertigo, consistent with the results of many studies (Morimoto et al., 2019; Toupet et al., 2019). Depression had no significant effect on the degree of vertigo in patients with AVS and CVS in the present study, although of course the effect of anxiety and depression on the severity of these two types of vertigo may not be reflected due to the small number of cases of AVS and CVS patients in the study.

Age is one of the principal risk factors for development of both vestibular syndrome and psychological disorders (Maarsingh et al., 2010). However, there was no significant difference in comorbid anxiety or depression between patients with vertigo of different ages (60 years and older compared with those under 60 years) in the present study. Dietzek et al. (2018) analyzed 650 patients with chronic vertigo who underwent multimodal vestibular rehabilitation and found that anxiety-related scores were lower in older patients over 65 years of age compared to young and middle-aged adults, and concluded that older adults are affected primarily by physical deficits and anxiety and other psychological factors are less influential. It was previously reported that female patients with vertigo are more likely to experience anxiety and depression than male patients, and that the degree of anxiety and depression in female patients is higher than in male patients. This may be associated with differences in brain structure and function between males and females (Asher and Aderka, 2018; Lindell et al., 2022). In the present study, the probability of anxiety and depression did not differ by gender among vertigo patients. This differs from previous studies and may be related to the small sample size of the present study; future studies will focus on large, multi-center samples of vertigo patients.

## 5. Limitation

This study compared the psychological factors of AVS, EVS, and CVS in patients with vestibular syndrome. In the same type of vertigo syndrome, the psychological manifestations of each classified diagnosis disease may differ, especially in EVS. In this study, the statistical analysis among classified diseases was limited due to the small number of cases in a few classified diseases. Also, the psychological status of different diagnosed diseases in the same syndrome was not studied extensively. In future studies, we will conduct more in-depth research and improve on this.

## 6. Conclusion

In this study, age and gender were not major contributors to a more severe course of vestibular syndromes. Instead, anxiety and depression were found to play a more prominent role. However, the comorbidity of anxiety and depression in patients with vertigo is often overlooked in clinical practice, which exacerbates subjective disability and diminishes patients’ quality of life. This highlights a crucial insight into managing vertigo: conducting a standardized psychological screening at the outset of treatment can effectively identify patients with comorbid anxiety/depression or other psychological factors, enabling the targeted implementation of psychological interventions. In this regard, scales such as GAD-7/PHQ-9 are valuable tools for rapidly screening patients with vertigo. Also, it is necessary to develop and implement a new screening tool that can assess more accurately the psychological distress experienced by patients with vertigo.

## Data availability statement

The raw data supporting the conclusions of this article will be made available by the authors, without undue reservation.

## Ethics statement

The studies involving human participants were reviewed and approved by the Medical Research Ethics Committee of the First Affiliated Hospital of China Medical University. Written informed consent to participate in this study was provided by the participants’ legal guardian/next of kin.

## Author contributions

SF designed the research. SF and JZ drafted the manuscript. JZ modified the manuscript. Both authors have read and approved to the final version of the manuscript.
