# Supplementary material for: The effect of accompanying anxiety and depression on patients with different vestibular syndromes
Source: Front Aging Neurosci. 2023 Aug 1;15:1208392. doi: 10.3389/fnagi.2023.1208392 (PMC10427919; doi:10.3389/fnagi.2023.1208392)
Supplement: Supplementary file 1 [file Table_1.DOCX]

Supplementary Material

The Effect of Accompanying Anxiety and Depression on Patients with Different Vestibular Syndromes

Shuai Feng，Jian Zang*

*** Correspondence:**Jian Zang: [jzang@cmu.edu.cn](mailto:jzang@cmu.edu.cn)


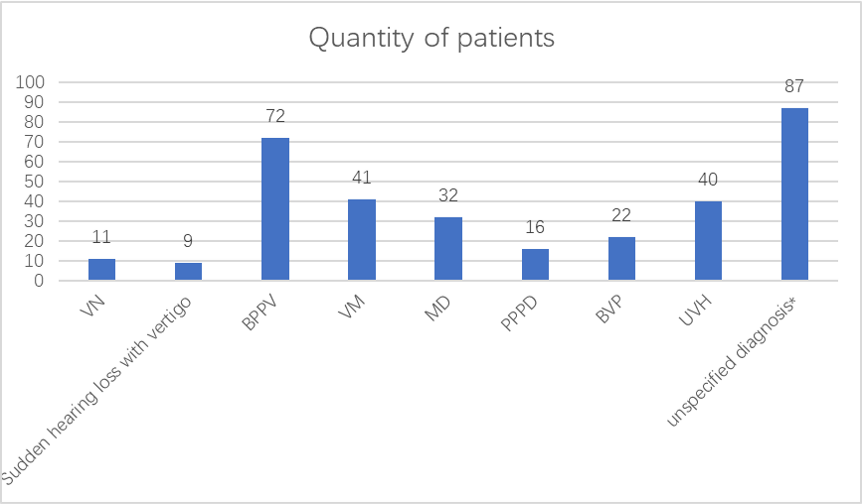


**Figure 1.** Quantitative distribution of each disease in total vertigo patients.

* The unspecified diagnosis included suspected VP, PCI, and TIA.
